# Fly Swatting

**Published:** 1913-06

**Authors:** 


### Fly Swatting.

As an epigrammatic expression of a general hygienic truth, the injunction to swat the fly has done good work. The public has learned just how dangerous and filthy the fly is—or rather it has received an exaggerated idea of its danger and filthiness for, like many other direct menaces to human health, the fly is dangerous mainly because he reflects man's own filthiness upon him. Perhaps it is well to leave the laity with a slightly exaggerated idea of this danger, as of many others. But it would be well to progress to the second lesson that "swat the fly" should be taken rather in a figurative than in a literal sense.

Fly swatting is all right as applied to the first ones of the season and to the occasional ones that pass by screens. A dead fly is, on some accounts, less dangerous than a live one, but is still a danger, perhaps more dangerous than the one that escapes from the house. If the fly is swatted and then burned or—with due disregard for the down stream population—dropped down a sewer, it is disposed of. If it is swatted and its carcass left in a corner of the living room it is only less dangerous than when flying about and a good deal more so than if driven out doors.

Fly swatting is also indirectly dangerous by detracting attention from the wholesale methods of screening, catching with papers, poisonous solutions, etc., and by the cardinal fact that it is not the fly itself but the germ-laden matter on which it feeds that is the ultimate source of danger. If there were no flies, open manure boxes would still be somewhat dangerous, and exposed privy vaults, garbage cans, sputum, etc., very dangerous. And, if the feeding places of flies, and especially those liable to special forms of contamination, were done away with, the fly itself would be almost harmless.

The particular danger of the slogan "Swat the fly" is that it is liable to be taken up by children, especially in the vacation season. Children are very impressionable, and they are likely to feel that a fly swatting campaign is a meritorious act and that the more flies they kill the better. Without entering into the question of the ethical influence on the child's psychology, of training it to kill anything, it is only necessary to point out that the child, impressed with the injunction to swat the fly, is going where there are the most flies to swat—in other words, just where the fly danger is particularly great. Children are not marked for aesthetic notions, nor for systematic concentration. Their fly-swatting campaigns about the manure pile, privy or garbage can, will be interrupted occasionally to get a drink of water, or a stick of candy, or for meals, and they will not always wash their hands.

If it were at all conceivable that the puny efforts of the little ones would accomplish as much against the fly as any one of many simple methods of prevention and exclusion, there might be something to be said in favor of entrusting the campaign to them. But, in the long run, teaching children to swat the fly will produce no appreciable hygienic effect except to magnify the dangers of the fly by intensifying the chances of infection among the least resistant members of the community.

